# Best Practice Guide for Cryoballoon Ablation in Atrial Fibrillation: The Compilation Experience of More than 1000 Procedures

**DOI:** 10.3390/jcdd10020055

**Published:** 2023-01-30

**Authors:** Dimitriοs Tsiachris, Christos-Konstantinos Antoniou, Ioannis Doundoulakis, Panagiota Manolakou, Demetrios Sougiannis, Athanasios Kordalis, Konstantinos A. Gatzoulis, Gian-Battista Chierchia, Carlo de Asmundis, Christodoulos Stefanadis, Konstantinos Tsioufis

**Affiliations:** 1 Athens Heart Centre Athens Medical Centre, 11526 Athens, Greece; 2 First Department of Cardiology, National and Kapodistrian University, Hippokration Hospital, 11527 Athens, Greece; 3 Heart Rhythm Management Centre, Universitair Ziekenhuis Brussel-Vrije Universiteit Brussel, 1090 Brussels, Belgium; 4 Section of Cardiovascular Medicine, Yale University School of Medicine, New Haven, CT 06510, USA

**Keywords:** atrial fibrillation, cryoballoon ablation, time to isolation, review

## Abstract

Nowadays, the cryoballoon (CB) constitutes an established alternative to radio frequency (RF) ablation for pulmonary vein isolation (PVI), which offers the possibility to isolate the PVs with a single application. Since the introduction of the second-generation CB, we prospectively collected our data to optimize the procedure on >1000 consecutive patients who underwent CB PVI performed in our center. It is expected that subsequent guidelines will suggest first-line PVI through CB in patients with paroxysmal AF with a class I indication. Indeed, in the long-term follow-up (36 months) of the EARLY-AF trial, CB had a lower incidence of persistent atrial fibrillation episodes compared to the anti-arrhythmic drugs group. We now review the current best practices in an effort to drive consistent outcomes and minimize complications. PV isolation through CB is the most studied single-shot technique for atrial fibrillation ablation, having shown the potential to alter the natural history of the arrhythmia. Several procedural tips and tricks can improve procedural flow and effectiveness. In the present article we provided not only technical details but measurable biophysical parameters that can reliably guide the operator into achieving the best outcome for his patients.

## 1. Introduction

Pulmonary vein isolation (PVI) represents the cornerstone of catheter atrial fibrillation (AF) ablation in symptomatic patients [1,2,3]. The second-generation cryoballoon (CB) (Arctic FrontTM, Medtronic Inc, Minneapolis, MN), released in 2012, achieved more uniform cooling across the entire distal hemisphere of the balloon using 8 instead of 4 injection tubes [4,5] considerably improving the acute results of PVI as well as the long-term efficacy in diverse clinical settings [6,7,8,9,10,11,12,13,14]. Nowadays, the CB constitutes an established alternative to radio frequency (RF) ablation for PVI [1,15], which offers the possibility to isolate the PVs with a single application.

Since the introduction of the second-generation CB, we prospectively collected our data to optimize the procedure on >1000 consecutive patients who underwent CB PVI performed in our center. Hence, we now review the current best practices in an effort to drive consistent outcomes and minimize complications.

## 2. Patient Selection for AF Ablation through Cryoablation

Ablation is recommended, in general, as a second-line therapy after failure (or intolerance) of class I or class III antiarrhythmic drugs in patients with paroxysmal or persistent AF through PVI either by means of CB or RF [1,2].

Several randomized controlled trials and observational studies have compared point-by-point RF and CB ablation, mostly in the first procedure for paroxysmal AF reporting broadly similar arrhythmia-free survival and overall complications with either technique, with slightly shorter procedure duration but longer fluoroscopy time with CB ablation [16,17,18]. However, some studies showed reduced hospitalization and lower complication rates with CB ablation [19,20,21]. It is important to identify specific indications for the preferential use of CB instead of RF for PVI in either paroxysmal or persistent AF.

### 2.1. Paroxysmal AF

The European Society of Cardiology and the major North American Society guidelines suggest first-line AF ablation only in highly selected patients with symptomatic paroxysmal (Class IIa) or persistent (Class IIb) AF without major risk factors for recurrence and considering patient preference, benefit and risk [1,2].

The above limited indication was based on studies employing first-line catheter ablation strategy using RF energy, which failed to demonstrate a significant improvement in clinical outcomes (45.5–52.7% freedom from atrial tachyarrhythmia in the ablation arm vs. 27.9–43.9% in the antiarrhythmic drug arm) and was characterized by high rates of cross-over and repeat ablation procedures [22,23,24].

Recently, three randomized, controlled trials compared CB ablation to antiarrhythmic drugs as first-line therapy of AF: the EARLY-AF (Early Aggressive Invasive Intervention for Atrial Fibrillation) trial [25]; the STOP-AF First (Cryoballoon Catheter Ablation in an Antiarrhythmic Drug Naive Paroxysmal Atrial Fibrillation) trial [26]; and the Cryo-FIRST (Catheter Cryoablation Versus Antiarrhythmic Drug as First-Line Therapy of Paroxysmal Atrial Fibrillation) trial [27]. These three randomized trials enrolled 724 highly symptomatic (mean Atrial Fibrillation Effect on QualiTy-of-life score 60.1) patients early in their disease course (median time from first AF diagnosis of 1 year), with 98% having paroxysmal AF (Table 1).

An initial treatment strategy of CB ablation in treatment-naive AF patients significantly reduced AF recurrence, improved symptoms and quality of life and did not increase adverse events compared with initial antiarrhythmic drug therapy [28]. It is expected that subsequent guidelines will suggest first-line PVI through CB in patients with paroxysmal AF with a class I indication [29,30].

Most recently, in the long-term follow up (36 months) of the EARLY-AF trial, CB had a lower incidence of persistent AF episodes compared to the AAD group (1.9 vs. 7.4%, HR 0.25). Recurrent atrial tachyarrhythmia was also significantly reduced in the 3-year follow up (56.5 vs. 77.2%, HR, 0.51), as well as the hospitalization rate (5.2% in the ablation group vs. 16.8% in the antiarrhythmic drug group, RR 0.31). Serious adverse events occurred in 7 patients (4.5%) in the ablation group and in 15 (10.1%) in the antiarrhythmic drug group [31].

### 2.2. Persistent AF

As the AF disease progresses, there is an increase of left atrial substrate involvement, greater left atrial wall fibrosis and left atrial dilation, greater proarrhythmic diversion circuits and less conductive healthy tissue [32]. Additional ablation beyond PVI has been examined such as linear lesions, complex fractionated atrial electrograms or rotors ablation, scar homogenization and non-PV triggers ablation failing to improve clinical outcomes [33,34,35,36,37,38].

Although, CB ablation is characterized by the inability to perform additional ablation (beyond superior vena cava and left atrial appendage isolation), a meta-analysis of 11 studies summarized data on the safety and mid-term efficacy of PVI using CB in patients with persistent AF, yielded a 68.9% freedom from recurrence after 1 year follow-up [39].

### 2.3. Heart Failure

The indispensability of AF ablation in the management of patients with concomitant heart failure has been both shown in landmark trials and confirmed in meta-analyses. Indeed, a recent review [40] reported that, in the population of heart failure patients at functional stages I–III, catheter ablation decreases all-cause mortality and improves all-atrial arrhythmia recurrence rate as well as cardiac function when compared to medical management—findings are still inconclusive regarding the sickest heart failure patients, at functional stage IV. Focusing on CB ablation, long-term outcomes on functional stage and ejection fraction appear favorable and equivalent to those achieved with the RF-based approach [41,42].

## 3. Preprocedural Imaging

To begin with, preprocedural imaging of the atria is of paramount importance to proper procedural planning and success, as made evident in the transseptal puncture section, inasmuch as it allows not only the visualization of the relative position of each vein (e.g., a highly cranial ostium) but also the assessment of the left atrium size, antral sizes and plan beforehand for any anatomical variations [43]. Thorax Coronary Tomography (CT) has indicated that here is considerable anatomic variability in pulmonary venous to left atrial drainage, as well as vein size and ovality [43,44]. Right PV ostia tend to be more circular and have greater orifice eccentricity than the left, with a significant difference in circularity between right- and left-sided veins, independently from the presence of AF or sinus rhythm [44,45]. Additionally, in a large cohort undergoing cardiac magnetic resonance angiography prior to initial AF ablation, over 5% of patients had at least one standard PV with a dimension greater than that of the CB (28 mm) [45].

Several reports have described acute success, AF recurrence and complications after CB ablation based on the PV anatomy using CT [43]. Regarding acute success and AF recurrences, thinner width of the left lateral ridge, higher PV ovality, PV ostium-bifurcation distance, shorter distance from the non-coronary cusp to inferior PVs, shallower angle of right PVs against the atrial septum and larger RSPV were associated with poor outcomes. Regarding complications, shorter distance between the RSPV ostium and the right phrenic (PN) nerve, larger RSPV-left atrium angle, larger RSPV area and smaller right carina width were associated with incidences of PN injury [44].

Presence of left common PV is crucial, and use of CB for PVI in this setting is controversial. Beiert et al. reported a 13.7% prevalence of left common PV. Patients with left common PV had significantly reduced AF-free survival after ablation with the second-generation CB (55.1% vs. 70.6%) [46]. In contrast, Stroker et al. reported success in isolating 139 left common PVs using the CB and demonstrated no difference in outcomes when compared to patients without LCPVs [47].

## 4. Procedural Tips and Tricks

- Deep sedation or general anesthesia

The best modality of sedation for AF ablation in general is still a matter of debate and center preference. Procedures are being performed with deep or conscious sedation or general anesthesia [48].

General anesthesia is accompanied by improved patient comfort and lack of patient movement allowing better catheter stability and lesion formation [49]. On the other hand, it is associated with increase in overall procedure time, intubation-related injuries, aspiration, or anaphylaxis with neuromuscular blocking drugs.

Focusing on CB ablation procedures, it has been exhibited that ablation with cryoenergy is significantly less painful compared to ablation with RF energy [50]. Pain reactions occur more frequently with RF ablation reducing catheter stability and increasing the risk of complications [50]. CB, therefore, may be considered for patients at a higher risk for anesthesia-related complications. On the other hand, deep or conscious sedation is preferred to local anesthesia, only since it had a better long-term outcome [51].

From a practical point of view, deep sedation is induced using boluses of midazolam and analgesics (phentanyl in our practice), while propofol administration may necessitate the presence of anesthesiologist due to either medical or legal reasons [48]. Our current approach is to administer a bolus of 2–5 mL of a 2% (20 mg/mL) propofol solution, followed by maintenance infusion at 15 mL/h, with additional 2 mL boluses administered and infusion rate increases based on clinical effect.

Invasive blood pressure measurement via a left radial artery peripheral catheter and oxygen saturation should be constantly monitored. Routine urinary catheterization with indwelling catheters is not advised in any form of patient sedation. External urinary catheters may be used according to operator discretion and to increase patient comfort.

General anesthesia might be preferred in cases of morbid obesity, severe obstructive sleep apnea syndrome and heart failure as well as in patients with a history of alcohol abuse in order to avoid paradoxical reactions after midazolam use with agitation, confusion and delirium.

- Femoral access

We perform two right-sided venous punctures: one for the transseptal sheath and one to introduce a steerable diagnostic multipolar catheter that is subsequently positioned in the coronary sinus and superior vena cava for PN stimulation. If available, ultrasound-guided venipuncture is preferred since it has been associated with a near-to-zero risk of vascular complications in patients undergoing CB ablation [52]. It is generally recommended to place the two sheaths in a certain distance in order to allow subsequent advancement of the FlexCath Advance Steerable Sheath.

- Transeptal access

Transseptal puncture is performed using a standard needle via a standard sheath. The transseptal needle is connected with a pressure monitor. We recommend a low and relatively anterior transseptal puncture as it offers greater mechanical advantages for the CB and Achieve mapping catheter when accessing PVs. Such a puncture allows more space for the CB to be rotated posteriorly to the right inferior PV also improving CB contact with inferior aspects of the inferior PV. Fluoroscopy and pressure monitoring are used to assist the transseptal puncture procedure. In difficult transseptal cases, use of transesophageal echocardiography or intracardiac echocardiography is recommended.

Arrive™ Braided Transseptal Sheath is preferred because it is larger and more hydrophilic allowing easier exchange with the FlexCath. When a more acute angle of attack is needed, such as in cases with distended atria, the operator may choose to either select a sheath with more pronounced curvature or to manually deform the needle into a more acute angle to achieve better apposition against the interatrial septum and thus produce the characteristic “bump” of the needle during retraction over the limbus. Occasional difficulties may be encountered pushing the FlexCath sheath across the septum. Placing the stiff exchange guidewire in the left superior PV or maneuvering the guidewire to the right superior PV will allow an easier push across the septum in a straighter manner. In obese patients and in cases of multiple previous femoral punctures, dilatation of the groin with surgical tools is crucial for FlexCath introduction.

- Technical tips and tricks for PVI using CB

Despite being more straightforward than the RF-based ablation approach, being a one-shot (for each vein) technique, there are several caveats where attention is necessary in order to ensure both proper placement of the lesion (line of block) and its durability [53,54].

➢ Approaching the veins

  i. As a general rule, depth of the sheath and/or balloon is assessed with the ipsilateral (relative to the target PVs) projection (e.g., left anterior oblique for the left PVs), providing a more longitudinal view of the vein taking into account the borders of the cardiac silhouette, whilst their orientation with regards to the frontal plane of each vein is assessed using the contralateral projection (e.g., right anterior oblique for the left PVs), where veins course relatively perpendicularly to the imaging plane [54].
 ii. It is extremely important to be mindful and to note the presence of antral potentials at the time of vein catheterization through the circular multipolar catheter (Achieve) since proper position of the CB may preclude such recording later during lesion formation (e.g., Achieve catheter may be needed to provide support and to be lodged distantly in the vein to ensure balloon stability).
iii. In order to transit from the left PVs to the right ones, the sheath (obviously with the balloon in the retracted position and the Achieve catheter projecting from its tip) should be flexed slightly rather than excessively in order to avoid missing the most cranial right PV. Given the more anteriorly placed transseptal puncture site used for PV isolation, to facilitate access to the right inferior PV, clockwise rotation is preferred in order to avoid scraping the anterior atrial wall.
 iv. In more horizontal orientation of the heart, as in obese individuals, it is likely that the most inferior right PV will apparently course behind the diaphragm, with the contrast appearing at the level of the liver (since the posterior basal-most part of the right inferior pulmonary lobe extends caudally to this level). If no such image is obtained during previous PV occlusions, the operator should try and map the area of interest and, once a vein with the proper contrast staining is located, either confirm its isolation (Achieve was placed in a more cranial branch during previous lesion delivery) or promptly isolate it.
  v. The PVs may be targeted either in the sequence of LSPV–LIPV–RSPV–RIPV or in a clockwise sequence (LSPV–LIPV–RIPV–RSPV).

➢ Occluding the veins

Following catheterization, with the Achieve catheter held in place (deep enough to ensure stability, regardless of potential recording, at this stage), the tip of CB is advanced until its distal marker is at the border of the cardiac silhouette in the ipsilateral projection and inflated under fluoroscopy.

**Figure 1:**
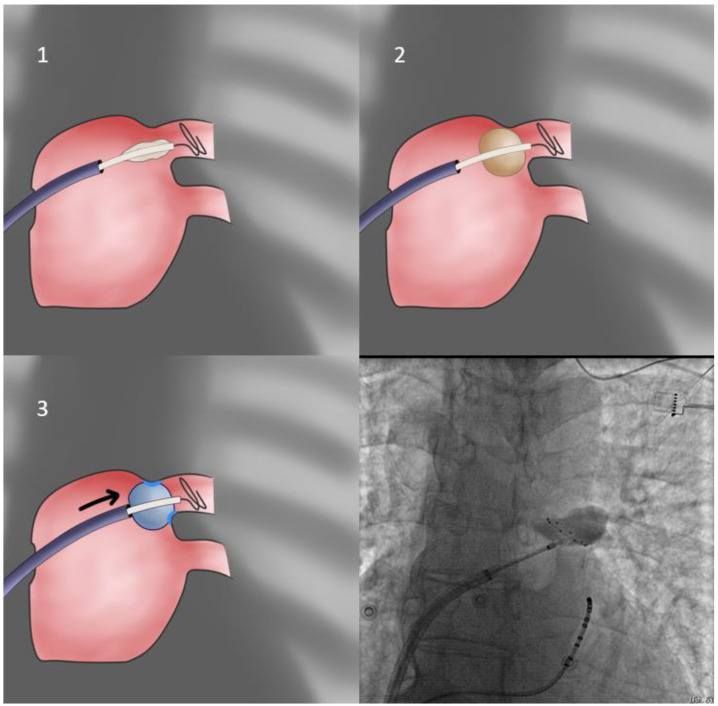
Direct approach. In this most basic technique, the catheter sheath is used to apply pressure to the balloon, leading to vein occlusion. Care must be taken to avoid pushing the balloon inside the venous ostium, leading to suboptimal isolation. Flexion of the balloon sheath itself may also be required (see text for further details).

1. Our first approach is using the sheath as the support for PV sealing (**FlexCath based direct approach**—Figure 1). With the whole system (Achieve catheter, cryoballoon and sheath) in contact and the sheath slightly flexed, we aim to align its distal part with the axis of the balloon and the Achieve catheter. Given that a vein may change the orientation of its course very near its antrum, it is the proximal part of the Achieve catheter that should be used for defining proper system alignment [53].

As mentioned, the longitudinal axis of the vein (using the proximal Achieve catheter as a surrogate) is evaluated through the ipsilateral projection, whilst the transverse one through the contralateral projection. When proper alignment is achieved, the FlexCath presses the CB against the antral wall. Contrast injection assists in evaluating proper sealing (circumferential occlusion) and, in cases of leakage, in assessing the location of the leak and adjusting orientation accordingly. The most common error amongst inexperienced operators at this point is overzealous application of force in the sheath, leading to improved sealing, at the cost of delivering an ostial, rather than antral, lesion, potentially leaving PV tissue proximally to the line of block. Consequently, the minimal pressure ensuring proper sealing in the selected orientation of the system must be applied. To verify this, the operator may, after achieving contrast medium pooling, slightly retract the system until leakage occurs at which point enough pressure to just contain it is reapplied.

**Figure 2:**
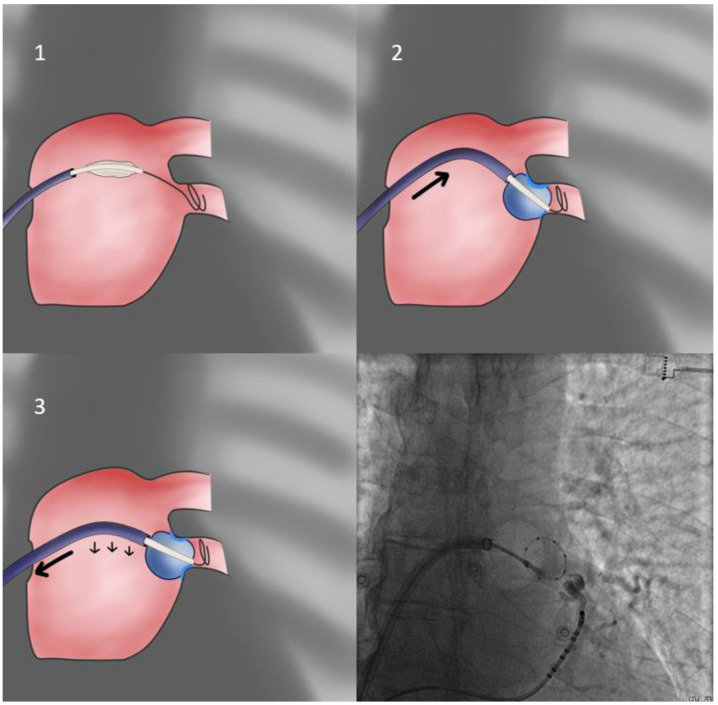
Hockey stick approach. This variation of the direct approach for the isolation of the inferior veins involves curving the catheter shaft more than in the direct approach, to take advantage of the more upward oriented inferior vein ostium orientation. Once proper alignment in the posterior/anterior plane is achieved (contralateral view is used—not shown) and the catheter, as well as the balloon, shafts are flexed, forward pressure leads to antral occlusion, with pressure mostly directed to the upper semicircle of the occlusion line. Consequently, after 60 s the pulldown maneuver is performed, with slight removal of flexion and gentle retraction of the catheter sheath to achieve greater contact force with the inferior part of the occlusion line circumference.

Regarding inferior veins with a descending course, the “**hockey stick**” formation of the system is often sought (Figure 2). In cases of highly angulated PVs, which the sheath flexion alone cannot accommodate, inputting additional caudal flexion of the CB catheter shaft is invaluable in achieving proper sealing. Crucially, the planes of flexion of the sheath and the CB shaft must coincide for an additive effect.

During the hockey stick approach in inferior PVs, it is likely that, upon applying pressure to the sheath and flexing the CB shaft, the inferior segment of the antrum will experience less pressure by the CB. Thus, it is advisable to perform a pulldown maneuver 60 s after lesion delivery initiation, consisting of slightly removing sheath flexion and, again slightly, retracting the sheath to obtain a more horizontal orientation, better pushing against said antral segment. Oftentimes, it is only after this maneuver is performed that potential elimination and vein isolation is observed.

**Figure 3:**
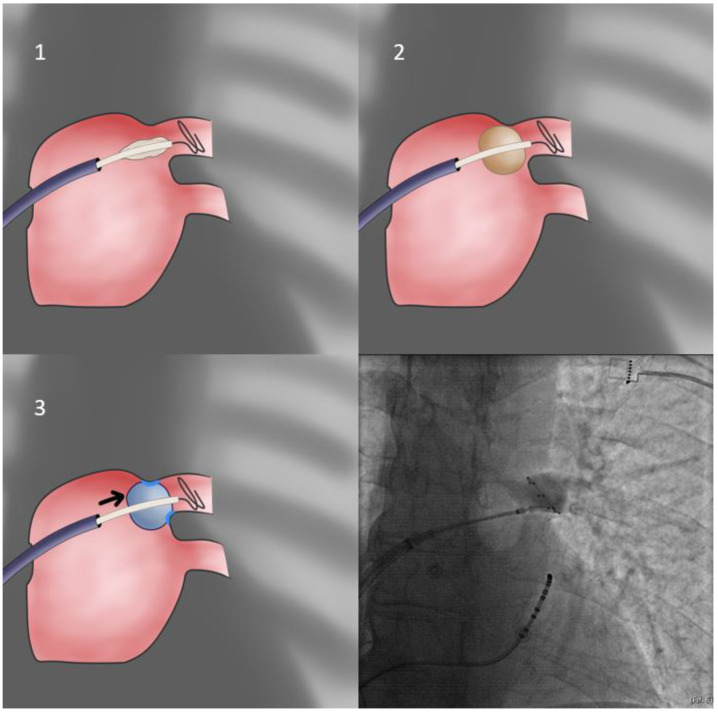
CB-only approach. In cases of unusually oriented veins, it is likely that pressure applied through the stiff catheter sheath will actually offset the balloon relative to the vein, preventing proper occlusion. Thus, the more flexible balloon shaft may be used as a more accommodating alternative. Balloon sheath flexion may or may not be helpful if this approach is chosen, depending on the particular anatomy of each case.

**Figure 4:**
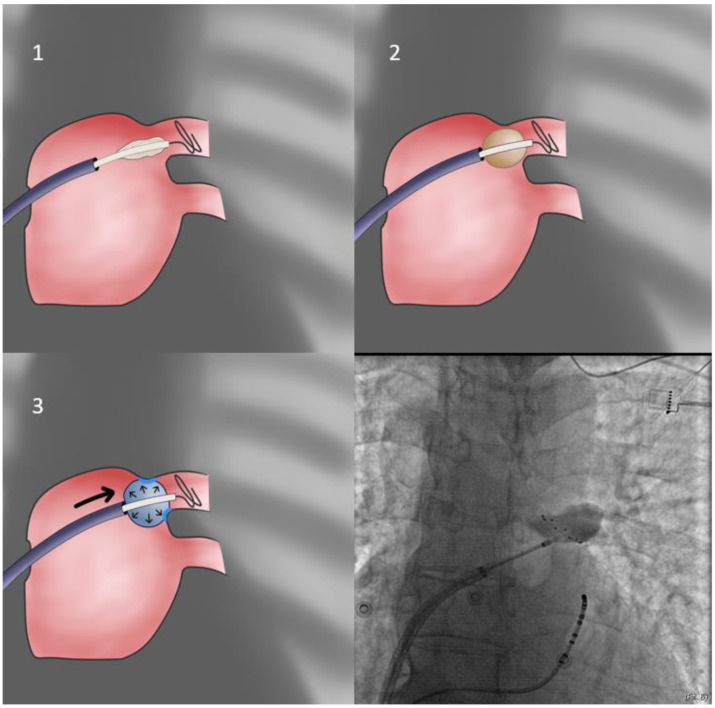
Delayed occlusion approach. This variation of the direct approach takes advantage of the slight increase in diameter of the balloon once it freezes. Thus, the operator will apply pressure when the temperature has started dropping below 20 °C, attempting to achieve better sealing. Otherwise, it is identical to the direct approach.

**Figure 5:**
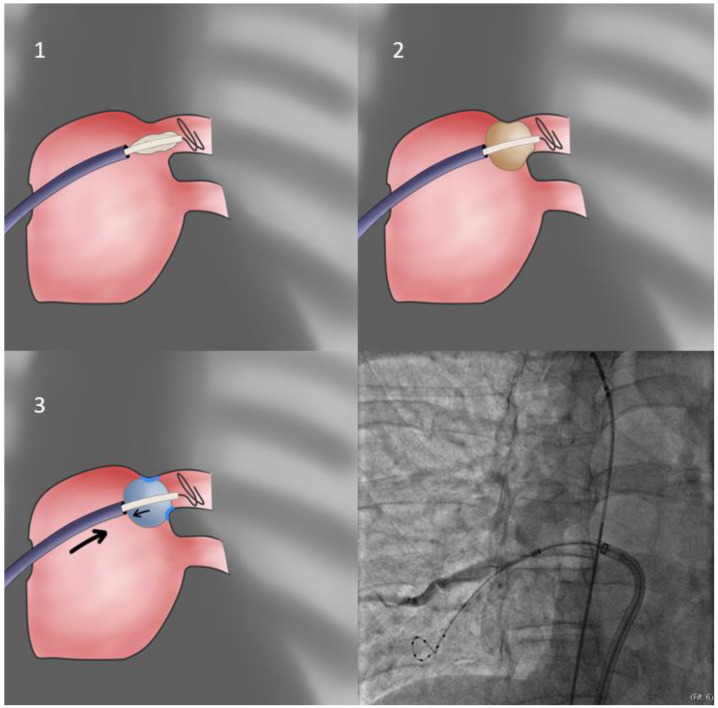
Trap-based approach. This is the most refined approach, mostly used for right inferior vein occlusion, although in principle, as shown, it can be used for all cases where achieving proper alignment of the catheter/balloon system with the pulmonary vein antrum is challenging. Thus, the operator attempts to use the pulmonary vein itself as a means to impart the proper alignment to the system by inflating the balloon slightly inside the vein. Then, as the balloon is ejected (care must be taken not to cause venous wall rupture by inflating too distally or by allowing for the fully pressurized balloon to dwell too long inside the vein—if not spontaneously ejected the balloon must be withdrawn). Then the closely positioned catheter sheath is promptly used to trap the balloon into place, which usually leads to adequate sealing of the vein. Again, it is emphasized that prior to inflation, proper orientation in the anterior/posterior plane must have been achieved, using the contralateral oblique projection.

2. In cases of cranial superior veins and horizontal inferior veins, we apply the **CB-only approach** (Figure 3) without the sheath (second approach). The sheath is relaxed and placed near but not in contact with the CB since even minimal flexion to the sheath will preclude proper sealing of the antrum. Selection of the more horizontal branch of the PV facilitates the CB-only approach, even if repositioning of the Achieve catheter is required. Additionally, cranial flexion of the CB shaft assists cranial superior PV sealing.

3. In cases of extremely sized antra and despite proper orientation, sealing is unattainable; the operator may elect to push the sheath forward only after freezing has begun, since freezing increases CB diameter from 26 to 28 mm, rendering it more likely to properly occlude the antrum (**delayed occlusion approach**—Figure 4). Usually, the cue for sheath pushing is the initiation of a steep temperature drop on the monitoring console screen, several seconds after freezing has commenced. In even rarer cases, where this approach is ineffective, and conversion to a point-by-point approach is undesirable, one may deliver lesions in a sequential manner, targeting each quadrant of the antrum separately, without occlusion, evaluating the effect after completion of the quartet of applications (segmental approach).

4. When, despite all efforts and maneuvers already described, the operator cannot achieve proper alignment of the Achieve/CB/sheath system with the vein, a final solution entails allowing the PV itself to position the CB in its antrum. This is achieved by purposefully and warily inflating slightly inside the PV ostium (obviously the operator must be alert and retract the system if severe CB distortion is observed), whilst the sheath is placed in the approximately anticipated orientation, slightly flexed in order to be rigid. It is expected that the PV will eject the inflating balloon while imparting its orientation to it—thus, the operator must be vigilant and immediately use the prepped sheath to intercept the motion and achieve apposition of the CB to the antrum in the vein-determined orientation (**trap-based approach**—Figure 5). If more pressure is needed to achieve occlusion, it is more prudent to use the balloon catheter shaft since pushing the rigid sheath may undo the achieved proper orientation of the system.

➢ Avoiding postablation PV stenosis

PV stenosis is a rather rare occurrence following introduction of the second-generation CB—mild stenosis (lumen narrowing 25–50% by multidetector computed tomography) was found to occur in 0.4% of PVs treated [55] and was associated with prolonged energy administration and wider initial diameter (often necessitating longer ablation times). However, in our experience, it is preventable by using certain simple principles. To begin with, in the ipsilateral view, no portion of the CB should be visible beyond the cardiac silhouette margins. In addition, the aforementioned slight sheath retraction after CB inflation may demonstrate intraluminal inflation, while simultaneously correcting its position. Careful assessment of balloon shape is a third visual cue to improper distal inflation. Finally, an unusually steep temperature drop, especially prior to the second coolant reservoir valve opening, with minimum values below −65 °C, may prompt a balloon position confirmation. In our experience, patient coughing is not evidence of too distal inflation, given that it is usually triggered by wall irritation by the Achieve catheter.

➢ Circular catheter maneuvers

Every effort must be made to obtain electrograms from the PVs by withdrawing the Achieve catheter and placing its tip as near the CB distal hemispherical surface as possible (pull-back maneuver) [45,46]. Usually this entails full withdrawal of the Achieve inside the CB catheter and slow re-advancement to attain a slanted orientation of the plane of the circular Achieve catheter relative to the vein (Flip-back maneuver). Clockwise/counterclockwise rotation of the Achieve may also be necessary during withdrawal or advancement to obtain proper contact. Based on the above, it is evident that the newer generation CB, with a shortened post-balloon tip, allows for more ease of placement of the Achieve catheter, while selection of the larger (20 mm vs. 15 mm) diameter of the latter also contributes to proper electrogram recording, even in larger veins. In certain cases, obtaining electrograms may necessitate repositioning of the system, even at the cost of seeking an alternative sealing position.

➢ Cryoapplications in right antra

Regarding the right PVs, more often than not, related to the presence of three rather than two right pulmonary lobes, there exists a significant branch of the superior or inferior vein (inferior/superior branch, respectively), if not an outright independent middle PV, deserving a dedicated occlusion and lesion delivery. Thus, best practice would dictate performing at least three applications of cryoenergy in the right side, ensuring altered system orientation and branch catheterization between lesion application in the two major PVs.

➢ Cryoapplications in left common PV

As mentioned above, there is rarely a single left common PV. In case its size is larger than the CB size, the segmental approach and the delayed occlusion approach may be used [52]. In case the CB seals the common PV, the operator should be aware that its occlusion equals simultaneously occluding both left PVs and thus reducing blood return to the heart by almost 50%. Hence, a significant drop in blood pressure should be expected. Moreover, the bronchial venous wall will be under significant tension, potentially leading to micro-ruptures and clinical hemoptysis.

➢ Cryoapplications in superior vena cava (SVC)

Should the operator choose to isolate the SVC, it is advisable to firstly verify that all relevant potentials are true SVC potentials, i.e., beyond the upper atrial limit, preventing any untoward effect to the sinoatrial node. This may be performed by occluding the SVC with the CB (confirmed by contrast retention) and examining whether the Achieve catheter records any signals. Subsequently, using the left anterior oblique view, the operator should flex the sheath and ensure its tip is pointing leftwards. Thus, upon pressure application, most of it will be directed away from the phrenic nerve, minimizing the likelihood for palsy to occur. Obviously, phrenic nerve stimulation is mandatory during SVC cryoapplication.

## 5. Cryoablation Dosing

Through the Achieve mapping catheter we are able to record the precise moment of PVI: this time-to-isolation (TTI) or time to effect denotes the elapsed time from the start of cryoapplication till PVI allowing distinguishing between efficient vs. inefficient applications [53,54,56].

In recent years, clinical and preclinical studies have evaluated the optimal CB dosing based on different TTI protocols with a focus on determining the shortest effective freezing duration [57,58,59,60,61,62,63]. TTI has emerged as a powerful marker of acute [57] and durable [58,59] PV isolation and has also been instrumental in reducing the need for the number of cryoapplications as well as the procedural duration and fluoroscopic utilization [62,63].

The impact of the diverse dose strategies was obvious in recent first line CB clinical trials. In the STOP-AF trial, TTI was not taken into consideration in order to assess acute efficacy and guide cryoenergy dose [26]. Given identical follow-up and similar baseline patients’ characteristics to the EARLY-AF trial, inter-study difference in success rate of the cryoablation (89% versus 74.6% regarding symptomatic episodes) may partially reflect suboptimal delivery of the invasive treatment in the STOP-AF. Inconsistently, ablation time was 3.1 min lower than expected (based on eight 3-min applications) and 5.2 min lower compared to EARLY-AF trial [25,26]. The implementation of the technique in low- volume centers (as 14 sites recruited 50 patients in total) might have also contributed to the substandard performance of cryotherapy.

A recent meta-analysis evaluated the effectiveness and safety of a TTI-based strategy of CB ablation in the treatment of AF and suggested that an individualized CB dosing strategy based on TTI and extended (greater than 2 min) duration of cryotherapy post TTI is accompanied by less recurrences post AF ablation. This is accomplished in the setting of a faster as well as safer procedure according to fewer PNP cases [64].

According to the abovementioned cryoablation index (TTI + 120 s), our proposed protocol, guided by more than 1000 performed procedures, was constructed in order to reduce complications and maximize effectiveness, incorporating nadir temperature as an additional parameter.

➢ TTI < 40 s and nadir temperature < −60 °C: total duration 180 s without bonus
➢ TTI 40–60 s and nadir temperature > −60 °C: total duration 240 s without bonus
➢ No TTI, temperature −40 °C within 60 s and nadir temperature < −60 °C total duration 180 s without bonus
➢ No TTI, temperature −40 °C within 60 s and nadir temperature > −60 °C total duration 240 s without bonus
➢ No TTI, temperature −40 °C > 60 s and nadir temperature > −60 °C total duration 240 s plus bonus

## 6. Predictors of PV Reconnection

One of the hottest issues in the field of AF ablation is PV isolation durability post RF, CB or pulsed field ablation. Several studies have analyzed data from redo electroanatomical mapping procedures post index CB ablation and have reported frequent PV sites of conduction recovery as well as having assessed procedural and biophysical indicators of PV reconnection (Table 2) [58,59,65,66,67,68,69,70,71,72].

In diverse multivariable analysis, colder nadir temperature, faster time to PV isolation and achievement of—40 °C within 60 s have been independently associated with durable PV isolation. The most frequent sites of reconnections were the superior-anterior portions for the upper PVs and the inferior-posterior portions for the lower PVs.

Based on our experience, from an initial cohort of 1000 CB ablations, a total of 42 patients (males: 85.7%; mean age: 59.8 +/− 11; EF 55.3% +/− 9.5; LAD 43.1 +/− 5 mm; CHADSVASC: 1.4+/−1.4) underwent a repeat ablation after 30.0 ± 18.3 months from the index CB ablation. All repeat ablations were performed using a 3-dimensional electro-anatomical mapping system. Among all 168 PVs, 35 (20.8%) showed a late PV reconnection in 22 patients, at the time of repeat ablation procedure. In 20 of 42 patients (47.6%), persistent isolation could be demonstrated in all PVs. Overall, persistent PV isolation could be documented in 133 of 168 PVs (79.2%). Right superior PV was persistently isolated in 73.8%, right inferior PV in 76.2%, left superior PV in 76.2% and left inferior PV in 90.5%. TTI was available in 73.2% of RSPVs, in 63.2% of RIPVs, in 70.7% of LSPVs and in 63.4% of LIPVs. In the univariate analysis failure to achieve TTI within 40 s (*p* = 0.04) was associated with late PV reconnection.

## 7. Complications

In general, complications rates are similar between ablation modalities (CB 5.4%, RF 5.2%, *p* = 0.806) [73], with a slight advantage for the former regarding cardiovascular complications (1.6% vs. 2.8%, *p* = 0.035, driven by decreases in post-ablation pericarditis, 0% vs. 0.6%, *p* = 0.008) and a slight disadvantage regarding phrenic nerve palsy incidence (1.5% vs. 0%, *p* < 0.001). All other complications, including tamponade, embolism, valvular injury, need for pacemaker implantation, atrioesophageal fistula, cerebrovascular events, access site-related and pulmonary (pneumothorax/hemothorax/PV stenosis) exhibited similar rates in both cohorts.

### 7.1. Atrioesophageal Fistula

The most dreaded postprocedural CB ablation complication, atrioesophageal fistula—mortality rates of 68.8% [74]—unfortunately remains somewhat of a mystery, not least given its rarity (incidence ranging from 0.00396% to 0.25% [74,75]. In CB ablation, it is mostly associated with lesions administered to the left inferior PV [76]; however, esophageal motility, even intraprocedurally, leads to potential association with lesions to any PV, except the right superior one [77]. The inciting event is, paradoxically, thought to be related to esophageal rather than atrial injury, due to direct thermal mucosal lesion, arteriole mechanical obstruction and increased gastroesophageal reflux due to damage to autonomic plexi controlling motility. No procedural parameters have been found to be predictive of its occurrence, and the only vague suggestion is to avoid prolonged ablation energy application to the posterior wall. This is inherently more difficult to achieve in CB ablation, given the simultaneous energy application to the ostium circumference. Monitoring of intraluminal esophageal temperature and mechanical displacement of the organ have been proposed as preventive measures but are still debated [75]. Proton pump inhibitor and H2 receptor blocker administration over a period of up to 6 weeks (4 weeks in our practice) is recommended. Given the exceedingly high mortality rate, patient education regarding the associated non-specific symptoms is likely the most significant life-saving measure.

### 7.2. Prevention of Phrenic Nerve Injury

Right PN palsy is the most common complication associated with CB ablation, and persistent PN injury has been reported as high as 8.3% [78]. Certain strategies have been proposed to minimize the risk for persistent PN injury.

- If neuromuscular blocking drugs have been administered during the induction of general anesthesia and sufficient time has not passed for the paralytic effect to reverse, neostigmine should be used.
- PN should be paced at the maximal output using a deflectable catheter placed above the level of the ablation in the junction of the superior vena cava and the right subclavian vein. Alternatively, the decapolar catheter can be placed in the anterolateral portion of the superior vena cava near the atrial–SVC junction.
- In case PN capture cannot be achieved or is not steady, the pacing dipole could be switched to the more distal (1 to 10 instead of 1 to 2 in the decapolar catheter).
- Palpation of the strength of diaphragmatic excursion during PN pacing, below the costal margin, is the most common method of monitoring PN function.
- Monitoring of the diaphragmatic compound motor action potential (CMAP), in addition to palpation, can increase the sensitivity of PN injury early detection [79]. Combining CMAP and palpation has decreased the incidence of PN injury to less than 1.5% [80].
- It is crucial to terminate ablation and deflate balloon immediately at the first sign of PN injury.
- In any case, CB should be positioned as antral as possible for PN injury avoidance.
- If recovery is fast and additional ablation is required, a different PV branch with a more antral position of the balloon should be preferred.
- In case PN injury persists the day after the procedure and confirmed by an inhalation–exhalation chest X-ray, physical therapy is essential with deep breathing exercises.

### 7.3. Postablation Pericarditis

Second-generation CB ablation has been associated with post-procedural acute pericarditis rates of 4% (although energy delivery protocol was more conservative than the one we propose) [81], with favorable outcomes in the overwhelming majority of cases (tamponade and mortality rates of 0.2% and 5%, respectively [82]). These are mainly attributed both to lesion formation and intracavitary catheter manipulation.

## 8. Post-Ablation and Post-Discharge Care

Postprocedural groin management also is not different than that for traditional RF ablation. An increased rate of groin access complication due to larger sheath size has not been reported. In our practice, guided by the more than 1000 performed procedures, we advise oral paracetamol use (max 4 g/24 h) as required, for chest pain management post-procedurally, and doctor notification if fever occurs in order for a short course of colchicine to be started. In general, these phenomena are limited to the first 3–4 days post-discharge. Use of corticosteroids, should be avoided, given their potential effect on lesion maturation and scarification of the atrial endocardium.

Same-day discharge might be a safe option, all the more appealing in the era of pandemics, provided certain prerequisites are met. These usually include [83] an uncomplicated procedure, successful deployment of a vascular closure device and adequate recovery, i.e., resumption of ambulation without issue. Using such a strategy has been proven both feasible (in >90% of cases) and safe (readmission rates at 30 days 3–4.8%) [84,85]. However, given that a recent study [85] reported an emergency department visit rate of 26.1%, similar for both RF and CB ablation, it should be advised to schedule a clinic visit within the first month post-discharge. 

A 2-month post-ablation anticoagulation duration is considered mandatory in all patients; this duration correlates with the complete tissue healing time observed in animal and in vivo experiences [1], given the lesser endocardium injury with cryoablation. Beyond this point, current anticoagulation guidelines should be used to determine need for long-term anticoagulation on a case-by-case basis.

There is not a suggested technique to prevent esophageal injury, and esophageal temperature cut-offs are not standardized. Therefore, high dose PPI is recommended in all patients 30 days post ablation for the prevention of esophageal fistula formation.

## 9. Conclusions

PV isolation through CB is the most studied single-shot technique for AF ablation, having shown the potential to alter the natural history of the arrhythmia. Several procedural tips and tricks can improve procedural flow and effectiveness. In the present article we provided not only technical details but measurable biophysical parameters, which can reliably guide the operator into achieving the best outcome for his patients.

## Figures and Tables

**Table 1:** The three randomized clinical trials showing superiority of pulmonary vein isolation with cryoballoon over antiarrhythmic therapy for prevention of recurrence in patients with paroxysmal atrial fibrillation.

	Patients (% Males)	Age	LAd (mm)	Monitoring Method	Cryo Protocol	Freedom from AF/AT (%)
EARLY AF [23]	303 (71%)	59	39	ECG ILR	(26.1), TTI	(89 vs. 73.8)—ECG (67.8 vs. 42.9)—ILR
STOP AF [24]	203 (59%)	61	38.5	24 h Holter	(20.9), no TTI	(74 vs. 45)
CRYO FIRST [25]	218 (68%)	52	38.5	7d Holter	Not standard	(82.2 vs. 67.6)

CRYO FIRST: Catheter Cryoablation Versus Antiarrhythmic Drug as First-Line Therapy of Paroxysmal Atrial Fibrillation, EARLY AF: Early Aggressive Invasive Intervention for Atrial Fibrillation trial, STOP AF First: Cryoballoon Catheter Ablation in Antiarrhythmic Drug Naive Paroxysmal Atrial Fibrillation, ECG: Electrocardiogram, ILR: Implantable loop recorder, LAd: Left atrial dimeter in mm, TTI: Time to isolation—see text for details.

**Table 2:** Studies that have analyzed data from redo electroanatomical mapping procedures post index CB ablation assessing procedural and biophysical indicators of PV reconnection.

	Redo Period	Patients	PVI	PVs Isolated	Predictors of Durability
Bordignon 2015 [56]	192 days	18	6	55/71	CB2 vs. CB1
Ciconte 2015 [58]	11.6 months	29	9	90/115	TTI < 60 s, Time to −40 °C < 60 s
Aryana 2016 [54]	14 months	112	41	324/435	TTI < 60 s, thawing time to 0 °C >10 s
Ciconte 2016 [57]	9.8 months	26	8	82/103	TTI, nadir T
Reddy 2015 [59]	3 months	21 (19)	15	68/75	Occlusion grade
Miyazaki 2016 [60]	6 months	40	15	119/158	Conduction delay
Miyazaki 2019 [61]	7 months	101	46	303/369	Nadir T, TTI, freezing and thawing speed
Scala 2020 [62]	18.8 months	148	68	377/487 (581)	Time to −40 °C, Nadir T, T at 60 s
Heeger 2015 [63]	205 days	66	17	178/258	Mean nadir T
Mugnai 2022 [64]	18.2 months	300	123	969/1178	Nadir T, TTI, time to −40 °C < 60 s

CB1: First generation cryoballoon, CB2: Second generation cryoballoon, PV: Pulmonary vein, PVI: Pulmonary vein isolation, T: Temperature, TTI: Time to isolation.

## Data Availability

Data available on the article.

## References

[1] Hindricks G., Potpara T., Dagres N., Arbelo E., Bax J.J., Blomström-Lundqvist C., Boriani G., Castella M., Dan G.A., Dilaveris P.E. (2021). 2020 ESC Guidelines for the diagnosis and management of atrial fibrillation developed in collaboration with the European Association for Cardio-Thoracic Surgery (EACTS): The Task Force for the diagnosis and management of atrial fibrillation of the European Society of Cardiology (ESC) Developed with the special contribution of the European Heart Rhythm Association (EHRA) of the ESC. Eur. Heart J..

[2] January C.T., Wann L.S., Calkins H., Chen L.Y., Cigarroa J.E., Cleveland J.C., Ellinor P.T., Ezekowitz M.D., Field M.E., Furie K.L. (2019). 2019 AHA/ACC/HRS focused update of the 2014 AHA/ACC/HRS guideline for the management of patients with atrial fibrillation: A Report of the American College of Cardiology/American Heart Association Task Force on Clinical Practice Guidelines and the Heart Rhythm Society. Heart Rhythm.

[3] Kawamura I., Neuzil P., Shivamurthy P., Kuroki K., Lam J., Musikantow D., Chu E., Turagam M.K., Minami K., Funasako M. (2021). How does the level of pulmonary venous isolation compare between pulsed field ablation and thermal energy ablation (radiofrequency, cryo, or laser)?. EP Eur..

[4] Knecht S., Kühne M., Osswald S., Sticherling C. (2014). Quantitative assessment of a second-generation cryoballoon ablation catheter with new cooling technology—A perspective on potential implications on outcome. J. Interv. Card. Electrophysiol..

[5] Coulombe N., Paulin J., Su W. (2013). Improved in vivo performance of second-generation cryoballoon for pulmonary vein isolation. J. Cardiovasc. Electrophysiol..

[6] Di Giovanni G., Wauters K., Chierchia G.-B., Sieira J., Levinstein M., Conte G., de Asmundis C., Baltogiannis G., Saitoh Y., Ciconte G. (2014). One-year follow-up after single procedure Cryoballoon ablation: A comparison between the first and second generation balloon. J. Cardiovasc. Electrophysiol..

[7] Aytemir K., Gurses K.M., Yalcin M.U., Kocyigit D., Dural M., Evranos B., Yorgun H., Ates A.H., Sahiner M.L., Kaya E.B. (2015). Safety and efficacy outcomes in patients undergoing pulmonary vein isolation with second-generation cryoballoon. EP Eur..

[8] Kumar N., Blaauw Y., Timmermans C., Pison L., Vernooy K., Crijns H. (2014). Adenosine testing after second-generation balloon devices (cryothermal and laser) mediated pulmonary vein ablation for atrial fibrillation. J. Interv. Card. Electrophysiol..

[9] Fürnkranz A., Bordignon S., Dugo D., Perotta L., Gunawardene M., Schulte-Hahn B., Nowak B., Schmidt B., Chun J.K.R. (2014). Improved 1-year clinical success rate of pulmonary vein isolation with the second-generation cryoballoon in patients with paroxysmal atrial fibrillation. J. Cardiovasc. Electrophysiol..

[10] Metzner A., Reissmann B., Rausch P., Mathew S., Wohlmuth P., Tilz R., Rillig A., Lemes C., Deiss S., Heeger C. (2014). One-year clinical outcome after pulmonary vein isolation using the second-generation 28-mm cryoballoon. Circ. Arrhythmia Electrophysiol..

[11] Jourda F., Providencia R., Marijon E., Bouzeman A., Hireche H., Khoueiry Z., Cardin C., Combes N., Combes S., Boveda S. (2015). Contact-force guided radiofrequency vs. second-generation balloon cryotherapy for pulmonary vein isolation in patients with paroxysmal atrial fibrillation-a prospective evaluation. EP Eur..

[12] Aryana A., Morkoch S., Bailey S., Lim H.W., Sara R., d’Avila A., O’Neill P.G. (2014). Acute procedural and cryoballoon characteristics from cryoablation of atrial fibrillation using the first- and second-generation cryoballoon: A retrospective comparative study with follow-up outcomes. J. Interv. Card. Electrophysiol..

[13] Chierchia G.B., Di Giovanni G., Ciconte G., de Asmundis C., Conte G., Sieira-Moret J., Rodriguez-Mañero M., Casado R., Baltogiannis G., Namdar M. (2014). Second-generation cryoballoon ablation for paroxysmal atrial fibrillation: 1-year follow-up. EP Eur..

[14] Ciconte G., de Asmundis C., Sieira J., Conte G., Di Giovanni G., Mugnai G., Saitoh Y., Baltogiannis G., Irfan G., Coutiño-Moreno H.E. (2015). Single 3-minute freeze for second-generation cryoballoon ablation: One-year follow-up after pulmonary vein isolation. Heart Rhythm.

[15] Iliodromitis K., Lenarczyk R., Scherr D., Conte G., Farkowski M.M., Marin F., Garcia-Seara J., Simovic S., Potpara T. (2022). Patient selection, peri-procedural management, and ablation techniques for catheter ablation of atrial fibrillation: An EHRA survey. Eurospace.

[16] Kuck K.H., Brugada J., Fürnkranz A., Metzner A., Ouyang F., Chun K.R., Elvan A., Arentz T., Bestehorn K., Pocock S.J. (2016). Cryoballoon or Radiofrequency Ablation for Paroxysmal Atrial Fibrillation. N. Engl. J. Med..

[17] Luik A., Radzewitz A., Kieser M., Walter M., Bramlage P., Hörmann P., Schmidt K., Horn N., Brinkmeier-Theofanopoulou M., Kunzmann K. (2015). Cryoballoon Versus Open Irrigated Radiofrequency Ablation in Patients With Paroxysmal Atrial Fibrillation: The Prospective, Randomized, Controlled, Noninferiority FreezeAF Study. Circulation.

[18] Neumann T., Kuniss M., Conradi G., Janin S., Berkowitsch A., Wojcik M., Rixe J., Erkapic D., Zaltsberg S., Rolf A. (2011). MEDAFI-Trial (Micro-embolization during ablation of atrial fibrillation): Comparison of pulmonary vein isolation using cryoballoon technique vs. radiofrequency energy. Europace.

[19] Kuck K.H., Fürnkranz A., Chun K.R., Metzner A., Ouyang F., Schlüter M., Elvan A., Lim H.W., Kueffer F.J., Arentz T. (2016). Cryoballoon or radiofrequency ablation for symptomatic paroxysmal atrial fibrillation: Reintervention, rehospitalization, and quality-of-life outcomes in the FIRE AND ICE trial. Eur. Heart J..

[20] Bollmann A., Ueberham L., Schuler E., Wiedemann M., Reithmann C., Sause A., Tebbenjohanns J., Schade A., Shin D.I., Staudt A. (2018). Cardiac tamponade in catheter ablation of atrial fibrillation: German-wide analysis of 21 141 procedures in the Helios atrial fibrillation ablation registry (SAFER). EP Eur..

[21] Ueberham L., Schuler E., Hindricks G., Kuhlen R., Bollmann A. (2018). SAFER. Eur. Heart J..

[22] Cosedis Nielsen J., Johannessen A., Raatikainen P., Hindricks G., Walfridsson H., Kongstad O., Pehrson S., Englund A., Hartikainen J., Mortensen L.S. (2012). Radiofrequency ablation as initial therapy in paroxysmal atrial fibrillation. N. Engl. J. Med..

[23] Morillo C.A., Verma A., Connolly S.J., Kuck K.H., Nair G.M., Champagne J., Sterns L.D., Beresh H., Healey J.S., Natale A. (2014). Radiofrequency ablation vs. antiarrhythmic drugs as first-line treatment of paroxysmal atrial fibrillation (RAAFT-2): A randomized trial. JAMA.

[24] Wazni O.M., Marrouche N.F., Martin D.O., Verma A., Bhargava M., Saliba W., Bash D., Schweikert R., Brachmann J., Gunther J. (2005). Radiofrequency ablation vs. antiarrhythmic drugs as first-line treatment of symptomatic atrial fibrillation: A randomized trial. JAMA.

[25] Andrade J.G., Wells G.A., Deyell M.W., Bennett M., Essebag V., Champagne J., Roux J.-F., Yung D., Skanes A., Khaykin Y. (2020). Cryoablation or Drug Therapy for Initial Treatment of Atrial Fibrillation. N. Engl. J. Med..

[26] Wazni O.M., Dandamudi G., Sood N., Hoyt R., Tyler J., Durrani S., Niebauer M., Makati K., Halperin B., Gauri A. (2021). Cryoballoon Ablation as Initial Therapy for Atrial Fibrillation. N. Engl. J. Med..

[27] Kuniss M., Pavlovic N., Velagic V., Hermida J.S., Healey S., Arena G., Badenco N., Meyer C., Chen J., Iacopino S. (2021). Cryoballoon ablation vs. antiarrhythmic drugs: First-line therapy for patients with paroxysmal atrial fibrillation. EP Eur..

[28] Kanagaratnam P., McCready J., Tayebjee M., Shepherd E., Sasikaran T., Todd D., Johnson N., Kyriacou A., Hayat S., Hobson N.A. (2022). Ablation versus anti-arrhythmic therapy for reducing all hospital episodes from recurrent atrial fibrillation: A prospective, randomized, multi-centre, open label trial. EP Eur..

[29] Andrade J.G., Wazni O.M., Kuniss M., Hawkins N.M., Deyell M.W., Chierchia G.B., Nissen S., Verma A., Wells G.A., Turgeon R.D. (2021). Cryoballoon Ablation as Initial Treatment for Atrial Fibrillation: JACC State-of-the-Art Review. J. Am. Coll. Cardiol..

[30] Leung L.W.M., Akhtar Z., Seshasai S.R.K., Gallagher M.M. (2022). First-line management of paroxysmal atrial fibrillation: Is it time for a ‘pill in the bin’ approach? A discussion on the STOP AF First, EARLY AF, Cryo-FIRST, and EAST-AF NET 4 clinical trials. EP Eur..

[31] Andrade J.G., Deyell M.W., Macle L., Wells G.A., Bennett M., Essebag V., Champagne J., Roux J.F., Yung D., Skanes A. (2022). Progression of Atrial Fibrillation after Cryoablation or Drug Therapy. N. Engl. J. Med..

[32] Walters T.E., Nisbet A., Morris G.M., Tan G., Mearns M., Teo E., Lewis N., Ng A., Gould P., Lee G. (2016). Progression of atrial remodeling in patients with high-burden atrial fibrillation: Implications for early ablative intervention. Heart Rhythm.

[33] Atienza F., Almendral J., Ormaetxe J.M., Moya A., Martínez-Alday J.D., Hernández-Madrid A., Castellanos E., Arribas F., Arias M., Tercedor L. (2014). Comparison of radiofrequency catheter ablation of drivers and circumferential pulmonary vein isolation in atrial fibrillation: A noninferiority randomized multicenter RADAR-AF trial. J. Am. Coll. Cardiol..

[34] Vogler J., Willems S., Sultan A., Schreiber D., Lüker J., Servatius H., Schäffer B., Moser J., Hoffmann B.A., Steven D. (2015). Pulmonary Vein Isolation Versus Defragmentation: The CHASE-AF Clinical Trial. J. Am. Coll. Cardiol..

[35] Verma A., Jiang C.Y., Betts T.R., Chen J., Deisenhofer I., Mantovan R., Macle L., Morillo C.A., Haverkamp W., Weerasooriya R. (2015). Approaches to catheter ablation for persistent atrial fibrillation. N. Engl. J. Med..

[36] Fink T., Schlüter M., Heeger C.H., Lemes C., Maurer T., Reissmann B., Riedl J., Rottner L., Santoro F., Schmidt B. (2017). Stand-Alone Pulmonary Vein Isolation Versus Pulmonary Vein Isolation With Additional Substrate Modification as Index Ablation Procedures in Patients With Persistent and Long-Standing Persistent Atrial Fibrillation: The Randomized Alster-Lost-AF Trial (Ablation at St. Georg Hospital for Long-Standing Persistent Atrial Fibrillation). Circulation. Arrhythmia Electrophysiol..

[37] Dohi T., Nakatani D., Inoue K., Hikoso S., Oka T., Hayashi K., Masuda M., Furukawa Y., Kawasaki M., Egami Y. (2019). Effect of Extensive Ablation on Recurrence in Patients with Persistent Atrial Fibrillation Treated with Pulmonary Vein Isolation (EARNEST-PVI) trial: Design and rationale. J. Cardiol..

[38] Lee J.M., Shim J., Park J., Yu H.T., Kim T.H., Park J.K., Uhm J.S., Kim J.B., Joung B., Lee M.H. (2019). The Electrical Isolation of the Left Atrial Posterior Wall in Catheter Ablation of Persistent Atrial Fibrillation. JACC Clin. Electrophysiol..

[39] Omran H., Gutleben K.J., Molatta S., Fischbach T., Wellmann B., Horstkotte D., Körber B., Nölker G. (2018). Second generation cryoballoon ablation for persistent atrial fibrillation: An updated meta-analysis. Clin. Res. Cardiol..

[40] Romero J., Gabr M., Alviz I., Briceno D., Diaz J.C., Rodriguez D., Patel K., Polanco D., Trivedi C., Mohanty S. (2022). Improved survival in patients with atrial fibrillation and heart failure undergoing catheter ablation compared to medical treatment: A systematic review and meta-analysis of randomized controlled trials. J. Cardiovasc. Electrophysiol..

[41] Rordorf R., Scazzuso F., Chun K.R.J., Khelae S.K., Kueffer F.J., Braegelmann K.M., Okumura K., Al-Kandari F., On Y.K., Földesi C. (2021). Cryoballoon Ablation for the Treatment of Atrial Fibrillation in Patients With Concomitant Heart Failure and Either Reduced or Preserved Left Ventricular Ejection Fraction: Results From the Cryo AF Global Registry. J. Am. Heart Assoc..

[42] Prabhu S., Ahluwalia N., Tyebally S.M., Dennis A.S.C., Malomo S.O., Abiodun A.T., Tyrlis A., Dhillon G., Segan L., Graham A. (2021). Long-term outcomes of index cryoballoon ablation or point-by-point radiofrequency ablation in patients with atrial fibrillation and systolic heart failure. J. Cardiovasc. Electrophysiol..

[43] Hayashi T., Murakami M., Saito S., Iwasaki K. (2022). Characteristics of anatomical difficulty for cryoballoon ablation: Insights from CT. Open Heart.

[44] Thorning C., Hamady M., Liaw J.V., Juli C., Lim P.B., Dhawan R., Peters N.S., Davies D.W., Kanagaratnam P., O’Neill M.D. (2011). CT evaluation of pulmonary venous anatomy variation in patients undergoing catheter ablation for atrial fibrillation. Clin. Imaging.

[45] Merchant F.M., Levy M.R., Iravanian S., Clermont E.C., Kelli H.M., Eisner R.L., El-Chami M.F., Leon A.R., Delurgio D.B. (2016). Pulmonary vein anatomy assessed by cardiac magnetic resonance imaging in patients undergoing initial atrial fibrillation ablation: Implications for novel ablation technologies. J. Interv. Card. Electrophysiol..

[46] Beiert T., Lodde P.C., Linneborn L.P.T., Werner J., Prinz L., Stöckigt F., Linhart M., Lickfett L., Nickenig G., Schrickel J.W. (2018). Outcome in patients with left common pulmonary vein after cryoablation with second-generation cryoballoon. Pacing Clin. Electrophysiol. PACE.

[47] Ströker E., Takarada K., de Asmundis C., Abugattas J.P., Mugnai G., Velagić V., de Regibus V., Coutiño H.E., Choudhury R., Iacopino S. (2017). Second-generation cryoballoon ablation in the setting of left common pulmonary veins: Procedural findings and clinical outcome. Heart Rhythm.

[48] Garcia R., Waldmann V., Vanduynhoven P., Nesti M., Jansen de Oliveira Figueiredo M., Narayanan K., Conte G., Guerra J.M., Boveda S., Duncker D. (2021). Worldwide sedation strategies for atrial fibrillation ablation: Current status and evolution over the last decade. EP Eur..

[49] Di Biase L., Conti S., Mohanty P., Bai R., Sanchez J., Walton D., John A., Santangeli P., Elayi C.S., Beheiry S. (2011). General anesthesia reduces the prevalence of pulmonary vein reconnection during repeat ablation when compared with conscious sedation: Results from a randomized study. Heart Rhythm.

[50] Attanasio P., Huemer M., Shokor Parwani A., Boldt L.H., Mügge A., Haverkamp W., Wutzler A. (2016). Pain Reactions during Pulmonary Vein Isolation under Deep Sedation: Cryothermal versus Radiofrequency Ablation. Pacing Clin. Electrophysiol. PACE.

[51] Chang T.Y., Lo L.W., Te A.L.D., Ishigaki S., Maesato A., Lin Y.J., Chang S.L., Hu Y.F., Chung F.P., Lin C.Y. (2021). Deep Sedation with Intravenous Anesthesia Is Associated with Outcome in Patients Undergoing Cryoablation for Paroxysmal Atrial Fibrillation. Int. Heart J..

[52] Ströker E., de Asmundis C., Kupics K., Takarada K., Mugnai G., De Cocker J., Stockman D., Sieira J., Schwagten B., Brugada P. (2019). Value of ultrasound for access guidance and detection of subclinical vascular complications in the setting of atrial fibrillation cryoballoon ablation. EP Eur..

[53] Bordignon S., Chen S., Bologna F., Thohoku S., Urbanek L., Willems F., Zanchi S., Bianchini L., Trolese L., Konstantinou A. (2021). Optimizing cryoballoon pulmonary vein isolation: Lessons from >1000 procedures- the Frankfurt approach. EP Eur..

[54] Su W., Kowal R., Kowalski M., Metzner A., Svinarich J.T., Wheelan K., Wang P. (2015). Best practice guide for cryoballoon ablation in atrial fibrillation: The compilation experience of more than 3000 procedures. Heart Rhythm.

[55] Coutiño H.E., Takarada K., Sieira J., Abugattas J.P., Salghetti F., De Regibus V., Ströker E., Brugada P., Iacopino S., de Asmundis C. (2018). Anatomical and procedural predictors of pulmonary vein stenosis in the setting of second-generation cryoballoon ablation. J. Cardiovasc. Med..

[56] Su W., Aryana A., Passman R., Singh G., Hokanson R., Kowalski M., Andrade J., Wang P. (2018). Cryoballoon Best Practices II: Practical guide to procedural monitoring and dosing during atrial fibrillation ablation from the perspective of experienced users. Heart Rhythm.

[57] Chun K.J., Bordignon S., Gunawardene M., Urban V., Kulikoglu M., Schulte-Hahn B., Nowak B., Schmidt B. (2012). Single transseptal big Cryoballoon pulmonary vein isolation using an inner lumen mapping catheter. Pacing Clin. Electrophysiol. PACE.

[58] Aryana A., Mugnai G., Singh S.M., Pujara D.K., de Asmundis C., Singh S.K., Bowers M.R., Brugada P., d’Avila A., O’Neill P.G. (2016). Procedural and biophysical indicators of durable pulmonary vein isolation during cryoballoon ablation of atrial fibrillation. Heart Rhythm.

[59] Ciconte G., Mugnai G., Sieira J., Velagić V., Saitoh Y., Irfan G., Hunuk B., Ströker E., Conte G., Di Giovanni G. (2015). On the Quest for the Best Freeze: Predictors of Late Pulmonary Vein Reconnections After Second-Generation Cryoballoon Ablation. Circ. Arrhythmia Electrophysiol..

[60] Chierchia G.B., de Asmundis C., Namdar M., Westra S., Kuniss M., Sarkozy A., Bayrak F., Ricciardi D., Casado-Arroyo R., Rodriguez Manero M. (2012). Pulmonary vein isolation during cryoballoon ablation using the novel Achieve inner lumen mapping catheter: A feasibility study. EP Eur..

[61] Kühne M., Knecht S., Altmann D., Ammann P., Schaer B., Osswald S., Sticherling C. (2013). Validation of a novel spiral mapping catheter for real-time recordings from the pulmonary veins during cryoballoon ablation of atrial fibrillation. Heart Rhythm.

[62] Chan N.Y., Yuen H.C., Chu P.S., Choy C.C., Chow H.F., Fong H.F., Lau C.L., Lo Y.K., Tsui P.T., Lau S.T. (2013). Inner lumen mapping catheter-facilitated big cryoballoon treatment for atrial fibrillation shortens procedural duration and fluoroscopic exposure with comparable mid-term efficacy. J. Interv. Card. Electrophysiol..

[63] Aryana A., Kowalski M., O’Neill P.G., Koo C.H., Lim H.W., Khan A., Hokanson R.B., Bowers M.R., Kenigsberg D.N., Ellenbogen K.A. (2016). Catheter ablation using the third-generation cryoballoon provides an enhanced ability to assess time to pulmonary vein isolation facilitating the ablation strategy: Short- and long-term results of a multicenter study. Heart Rhythm.

[64] Tsiachris D., Doundoulakis I., Antoniou C.K., Pagkalidou E., Zafeiropoulos S., Kordalis A., Gatzoulis K.A., Chierchia G.B., de Asmundis C., Tsioufis K. (2022). Effectiveness and safety of a time to isolation strategy of cryoballoon ablation of atrial fibrillation: A systematic review and meta-analysis. J. Cardiovasc. Electrophysiol..

[65] Bordignon S., Fürnkranz A., Perrotta L., Dugo D., Konstantinou A., Nowak B., Schulte-Hahn B., Schmidt B., Chun K.R. (2015). High rate of durable pulmonary vein isolation after second-generation cryoballoon ablation: Analysis of repeat procedures. EP Eur..

[66] Ciconte G., Velagić V., Mugnai G., Saitoh Y., Irfan G., Hunuk B., Ströker E., Conte G., Sieira J., Di Giovanni G. (2016). Electrophysiological findings following pulmonary vein isolation using radiofrequency catheter guided by contact-force and second-generation cryoballoon: Lessons from repeat ablation procedures. EP Eur..

[67] Reddy V.Y., Sediva L., Petru J., Skoda J., Chovanec M., Chitovova Z., Di Stefano P., Rubin E., Dukkipati S., Neuzil P. (2015). Durability of Pulmonary Vein Isolation with Cryoballoon Ablation: Results from the Sustained PV Isolation with Arctic Front Advance (SUPIR) Study. J. Cardiovasc. Electrophysiol..

[68] Miyazaki S., Taniguchi H., Hachiya H., Nakamura H., Takagi T., Hirao K., Iesaka Y. (2016). Clinical recurrence and electrical pulmonary vein reconnections after second-generation cryoballoon ablation. Heart Rhythm.

[69] Miyazaki S., Kajiyama T., Watanabe T., Nakamura H., Hachiya H., Tada H., Iesaka Y. (2020). Predictors of durable pulmonary vein isolation after second-generation cryoballoon ablation with a single short freeze strategy—Different criteria for the best freeze of the 4 individual PVs. Int. J. Cardiol..

[70] Scala O., Borio G., Paparella G., Varnavas V., Ströker E., Guimaraes Osorio T., Terasawa M., Sieira J., Maj R., Rizzo A. (2020). Predictors of durable electrical isolation in the setting of second-generation cryoballoon ablation: A comparison between left superior, left inferior, right superior, and right inferior pulmonary veins. J. Cardiovasc. Electrophysiol..

[71] Heeger C.H., Wissner E., Mathew S., Deiss S., Lemes C., Rillig A., Wohlmuth P., Reissmann B., Tilz R.R., Ouyang F. (2015). Once Isolated, Always Isolated? Incidence and Characteristics of Pulmonary Vein Reconduction After Second-Generation Cryoballoon-Based Pulmonary Vein Isolation. Circ. Arrhythmia Electrophysiol..

[72] Mugnai G., Cecchini F., Stroker E., Paparella G., Iacopino S., Sieira J., De Greef Y., Tomasi L., Bolzan B., Bala G. (2022). Durability of pulmonary vein isolation following cryoballoon ablation: Lessons from a large series of repeat ablation procedures. Int. J. Cardiology. Heart Vasc..

[73] Mörtsell D., Arbelo E., Dagres N., Brugada J., Laroche C., Trines S.A., Malmborg H., Höglund N., Tavazzi L., Pokushalov E. (2018). Cryoballoon vs. radiofrequency ablation for atrial fibrillation: A study of outcome and safety based on the ESC-EHRA atrial fibrillation ablation long-term registry and the Swedish catheter ablation registry. EP Eur..

[74] Piccini J.P., Braegelmann K.M., Simma S., Koneru J.N., Ellenbogen K.A. (2020). Risk of atrioesophageal fistula with cryoballoon ablation of atrial fibrillation. Heart Rhythm O2.

[75] Kapur S., Barbhaiya C., Deneke T., Michaud G.F. (2017). Esophageal Injury and Atrioesophageal Fistula Caused by Ablation for Atrial Fibrillation. Circulation.

[76] John R.M., Kapur S., Ellenbogen K.A., Koneru J.N. (2017). Atrioesophageal fistula formation with cryoballoon ablation is most commonly related to the left inferior pulmonary vein. Heart Rhythm.

[77] Pappone C., Vicedomini G., Santinelli V. (2013). Atrio-Esophageal Fistula After AF Ablation: Pathophysiology, Prevention &Treatment. J. Atr. Fibrillation.

[78] Packer D.L., Kowal R.C., Wheelan K.R., Irwin J.M., Champagne J., Guerra P.G., Dubuc M., Reddy V., Nelson L., Holcomb R.G. (2013). Cryoballoon ablation of pulmonary veins for paroxysmal atrial fibrillation: First results of the North American Arctic Front (STOP AF) pivotal trial. J. Am. Coll. Cardiol..

[79] Lakhani M., Saiful F., Parikh V., Goyal N., Bekheit S., Kowalski M. (2014). Recordings of diaphragmatic electromyograms during cryoballoon ablation for atrial fibrillation accurately predict phrenic nerve injury. Heart Rhythm.

[80] Mondésert B., Andrade J.G., Khairy P., Guerra P.G., Dyrda K., Macle L., Rivard L., Thibault B., Talajic M., Roy D. (2014). Clinical experience with a novel electromyographic approach to preventing phrenic nerve injury during cryoballoon ablation in atrial fibrillation. Circ. Arrhythmia Electrophysiol..

[81] Mugnai G., de Asmundis C., Iacopino S., Ströker E., Longobardi M., Negro M.C., De Regibus V., Coutino-Moreno H.E., Takarada K., Choudhury R. (2018). Acute pericarditis following second-generation cryoballoon ablation for atrial fibrillation. J. Interv. Card. Electrophysiol..

[82] Cappato R., Calkins H., Chen S.A., Davies W., Iesaka Y., Kalman J., Kim Y.H., Klein G., Natale A., Packer D. (2011). Delayed cardiac tamponade after radiofrequency catheter ablation of atrial fibrillation: A worldwide report. J. Am. Coll. Cardiol..

[83] Portoles-Hernandez A., Toquero-Ramos J., Garcia-Gomez S., Castro-Urda V., Garcia-Izquierdo E., Jimenez-Sanchez D., Segura-Dominguez M., Aguilera-Agudo C., Veloza-Urrea D., Fernandez-Lozano I. (2021). Same-day discharge for atrial fibrillation ablation: Use of suture-mediated vascular closure device. EP Eur..

[84] Rashedi S., Tavolinejad H., Kazemian S., Mardani M., Masoudi M., Masoudkabir F., Haghjoo M. (2022). Efficacy and safety of same-day discharge after atrial fibrillation ablation: A systematic review and meta-analysis. Clin. Cardiol..

[85] Deyell M.W., Hoskin K., Forman J., Laksman Z.W., Hawkins N.M., Bennett M.T., Yeung-Lai-Wah J.A., Chakrabarti S., Krahn A.D., Andrade J.G. (2022). Same-day discharge for atrial fibrillation ablation: Outcomes and impact of ablation modality. EP Eur..

